# Promiscuous Inflammasomes: The False Dichotomy of RNA/DNA Virus-Induced Inflammasome Activation and Pyroptosis

**DOI:** 10.3390/v14102113

**Published:** 2022-09-23

**Authors:** Hannah L. Wallace, Rodney S. Russell

**Affiliations:** Immunology and Infectious Diseases, Division of Biomedical Sciences, Faculty of Medicine, Memorial University, St. John’s, NL A1C 5S7, Canada

**Keywords:** inflammasome, pyroptosis, NLRP3, AIM2, virus-induced pyroptosis, inflammasome activation

## Abstract

It is well-known that viruses activate various inflammasomes, which can initiate the programmed cell death pathway known as pyroptosis, subsequently leading to cell lysis and release of inflammatory cytokines IL-1β and IL-18. This pathway can be triggered by various sensors, including, but not limited to, NLRP3, AIM2, IFI16, RIG-I, and NLRC4. Many viruses are known either to activate or inhibit inflammasomes as a part of the innate immune response or as a mechanism of pathogenesis. Early research in the field of virus-induced pyroptosis suggested a dichotomy, with RNA viruses activating the NLRP3 inflammasome and DNA viruses activating the AIM2 inflammasome. More recent research has shown that this dichotomy may not be as distinct as once thought. It seems many viruses activate multiple inflammasome sensors. Here, we detail which viruses fit the dichotomy as well as many that appear to defy this clearly false dichotomy. It seems likely that most, if not all, viruses activate multiple inflammasome sensors, and future research should focus on expanding our understanding of inflammasome activation in a variety of tissue types as well as virus activation of multiple inflammasomes, challenging biases that stemmed from early literature in this field. Here, we review primarily research performed on human viruses but also include details regarding animal viruses whenever possible.

## 1. Introduction

### 1.1. Inflammasome Activation/Pyroptosis

The first report of what is now known as pyroptosis was a study published in 1997 by Hilbi et al., which showed activation of caspase-1 in macrophages infected with *Shigella flexneri* [1]. The term “pyroptosis“ to describe caspase-1-mediated cell death was proposed for the first time in 2001 by Cookson and Brenna. They described pyroptosis as a form of programmed cell death that morphologically resembled necrosis, but was mediated by caspase-1, making it distinct from caspase-3-mediated apoptosis [2]. Early understanding of virus-induced inflammasome activation/pyroptosis leaned heavily towards understanding that the inflammasome was necessary for an adaptive immune response and necessary for clearing viral infections [3,4,5] (and reviewed in [6]). More recently, the role of the inflammasome during viral infection was re-evaluated, driving speculation that pyroptosis could be the leading factor behind deadly inflammatory responses associated with highly pathogenic viral infections, including pandemic-causing SARS-CoV-2 (severe acute respiratory syndrome coronavirus 2) [7,8,9,10].

**Figure 1:**
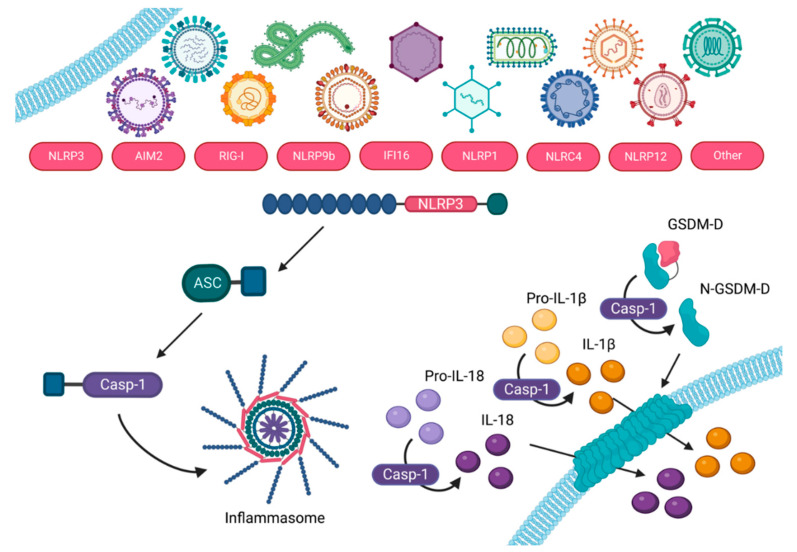
Virus-induced inflammasome pathway which ultimately results in pyroptosis. Numerous viruses can activate various inflammasome sensors such as NLRP3, AIM2. RIG-I, NLRP9b, IFI16, NLRP1, NLRC4, NLRP12, and/or others, directly or indirectly. The activated sensor then oligomerizes with ASC (if it does not contain its own CARD). Caspase-1 is then recruited to the inflammasome to act as the effector enzyme. Caspase-1 cleaves GSDM-D and the N-terminus migrates to the cellular membrane to form pores. At the same time, pro-IL-18 and pro-IL-1β are also cleaved by caspase-1 into their mature forms, and subsequently released through the pores formed by GSDM-D. This figure was created with BioRender.com and adapted from Jorgensen et al. [28].

Pyroptosis is an inflammatory form of programmed cell death mediated by a protein complex, dubbed the inflammasome [11], which contains a receptor such as NLRP3 (nucleotide oligomerization domain, leucine-rich repeat (NLR), pyrin-domain containing protein 3; [12]), AIM2 (absent in melanoma 2), IFI16 (interferon gamma inducible protein 16), or NLRC4 (NLR family, CARD domain containing 4), among others (reviewed in [13]), an adaptor protein, which is often ASC (apoptosis-associated speck-like protein containing a CARD (caspase recruitment domain)), unless the sensor contains its own CARD, and effector enzyme, caspase-1. Pyroptosis ultimately leads to pore formation by gasdermin-D (GSDM-D) in the cell membrane, causing cell swelling and subsequent lysis. This is accompanied by maturation and ensuing release of inflammatory cytokines IL-1*β* and IL-18 (extensively reviewed in reference [14]; Figure 1). Pyroptosis has been implicated in inflammatory pathogenesis induced by viruses [15,16,17,18,19,20,21,22] as well as being shown to protect the host as a necessary component of an effective adaptive immune response [23,24,25,26,27]. Whether pyroptosis is a mechanism of pathogenesis or a pathway necessary for an effective adaptive immune response in the context of viral infections remains generally unclear.

### 1.2. Virus-Induced Pyroptosis

The phenomenon of viruses activating inflammasomes, leading to inflammatory pyroptosis, is both well documented for some viruses and absent for others. Pyroptosis in the context of some viral infections, such as influenza, has been extensively studied (reviewed in [29,30]) while a simple search for other viruses and pyroptosis-related terms yield no peer-reviewed publications. Overall, there remain important questions to be answered. These include identifying the trigger of inflammasome activation/pyroptosis, determining whether certain sensors are only activated by specific kinds of viruses, and how pyroptosis is advantageous or detrimental to the virus and/or host.

### 1.3. The RNA/DNA Inflammasome Sensor Dichotomy

Early literature regarding virus-induced pyroptosis reported results that seemed to suggest that RNA and DNA viruses activate different sensors to trigger the inflammasome. This literature largely suggested a dogma by which RNA viruses activate the NLRP3 inflammasome [31] while the AIM2 or IFI16 inflammasome is activated by DNA viruses [32]. While this statement is not untrue, it is an overgeneralization that does not incorporate research suggesting the RNA-NLRP3 and DNA-AIM2/IFI16 dichotomy is inconsistent [20,23,33,34,35,36,37,38].

### 1.4. The Many Faces of the NLRP3 Inflammasome

Both AIM2 and IFI16 contain an HIN (hematopoietic expression, interferon-inducible nature, and nuclear localization) domain that can directly bind to double-stranded DNA, leading to logical early conclusions about their activation [39,40]. However, a clear understanding of what activates the NLRP3 inflammasome is lacking. There have been many hypotheses and much speculation, including, but not limited to, mitochondrial DNA [41,42], reactive oxygen species (ROS) [43,44], viral RNA [45,46,47], inflammasome complexes released from other cells [48,49], ATP [50,51], specific viral proteins [17,35,52,53,54,55], changes in ion concentrations such as potassium efflux [56,57], binding of/interaction with NEK7 [58,59], or even changes in cell volume [60]. To date, none of these hypotheses have been confirmed in all circumstances, leaving questions regarding whether there exists one specific trigger or multiple that activate NLRP3.

While, at first glance, inflammasome sensors seem very specific with regard to activation by viruses, there are hints throughout the literature of more promiscuous activity than was described in early reports [31,32]. There is a growing body of research suggesting that some viruses, including, but not limited to, herpes simplex virus 1(HSV-1) [34,61,62,63,64], human bocavirus 1 (HBoV1) [33,65], hepatitis B virus (HBV) [47,53,66,67,68,69], human adenovirus 5 (Ad5) [36,70,71,72], Zika virus [37], West Nile virus (WNV) [73,74,75,76], enterovirus A71 (EV-A71) [23,77], Mayaro virus (MAYV) [20], chikungunya virus (CHIKV) [76,78], influenza A virus (IAV) [5,15,16,17,25,26,38,79,80,81,82,83,84,85,86,87], SARS-CoV-2 [10,88], and human immunodeficiency virus (HIV) [89,90,91,92,93], activate multiple inflammasomes, particularly when one sensor is blocked or inhibited [20,22,37,38,66,67,72,79,80].

### 1.5. NLRP3 Is Triggered by WHAT? 

In the minds of the authors, there remain two primary outstanding questions in the field: what triggers NLRP3 and is there really a set RNA/DNA dogma? These questions may seem separate. However, some part or product from a given virus or cellular damage as a byproduct of viral infection likely triggers the inflammasome, suggesting involvement of viral nucleic acids, either entire viral genomes or replication intermediates, specific viral proteins, cytosolic sensing of mitochondrial DNA, or damage to the endoplasmic reticulum. The exact trigger of NLRP3 awaits conclusive research.

In general, research on inflammasome activation has been focused on this process in immune cells. However, more recent work has highlighted pyroptosis induction in a multitude of cell types, including, but not limited to, cardiomyocytes [43], hepatocytes [22,94], intestinal epithelia [95,96], neurons [97,98], airway epithelial cells [33], and tubular epithelial cells of the kidney [99]. The identification and characterization of inflammasome activation in tissue and cell types other than lymphocytes remains an exciting and somewhat unexplored topic that will undoubtedly expand our understanding of the inflammasome pathway.

## 2. Viruses That Fit the Dogma

### 2.1. DNA Viruses That Activate DNA-Associated Sensors

#### 2.1.1. Poxviridae

One early report of AIM2 activation in response to a DNA virus is a 2009 study by Hornung et al., which, importantly, identified AIM2 as a cytosolic DNA sensor that can form an inflammasome with ASC and regulate caspase-1 activation. By knocking down AIM2, the authors showed significantly decreased activation of caspase-1 in response to infection with vaccinia virus [39].

#### 2.1.2. Papillomaviridae

Pontillo et al. showed certain SNPs in inflammasome-associated genes (NLRP1, NLRP3, IL-18, and IL-1β) were linked to protection against human papillomavirus persistence and cervical cancer progression [100]. Reinholtz et al. detailed AIM2 and IFI16 activation in keratinocytes upon infection with human papillomavirus 16 [101] while work by Song et al. detailed how HPV E7 can inhibit IFI16-associated pyroptosis by promoting TRIM21-mediated degradation of the inflammasome [102]. Conclusive effects of the inflammasome on HPV infection remain to be elucidated.

#### 2.1.3. Herpesviridae

The majority of the adult human population is infected with human cytomegalovirus (HCMV) [103]. Since there is an excellent mouse model available, CMV has been well studied using mouse CMV (mCMV) as a proxy for HCMV. A study by Rathinam et al. sought to confirm the role of AIM2 in the regulation of caspase-1 activity and maturation of IL-1β and IL-18 in the context of CMV infection. The authors showed AIM2 was essential for inflammasome activation by mCMV as well as vaccinia virus. Production of IL-18 and interferon (IFN)-γ for fighting cytoplasmic viral infections was dependent on AIM2 activation during mCMV infection in mice [32].

Kaposi sarcoma-associated herpesvirus (KSHV) is associated with tumors characterized by inflammatory microenvironments, including high levels of IL-1β. Kerur et al. showed that KSHV infection of endothelial cells triggers IFI16 to interact with ASC, forming an inflammasome dependent on KSHV gene expression and/or latent KSHV genome presence. The inflammasome was initially located in the nucleus and subsequently moved to the perinuclear area. The IFI16 inflammasome co-localized with the KSHV genome in the nucleus of infected cells. Further, downstream activation of caspase-1 was inhibited by IFI16 and/or ASC silencing [104].

### 2.2. RNA Viruses That Activate NLRP3

#### 2.2.1. Flaviviridae

We and others have previously reported that hepatitis C virus (HCV) activates the NLRP3 inflammasome and subsequently induces pyroptosis [22,94,105,106]. Data from our most recent publication suggest inflammasome activation/pyroptosis may even contribute to the pathogenesis associated with HCV infection, as lower extracellular infectious titre was seen in cells with inflammasome pathway components knocked out [22]. Daussy et al. showed that the Golgi fragmentation associated with HCV infection is induced by the NLRP3 inflammasome [107]. Interestingly, these authors found the same effect for cells treated with nigericin but not when cells were infected with related flavivirus, Zika virus [107]. Noteworthy is the finding that levels of pyroptosis-associated cytokines Il-18 and IL-1β are increased in serum of individuals infected with HCV, even after curative therapy [108,109,110]. HCV may eventually be added to the list of viruses that violate the dichotomy as our most recent work also showed that CRISPR knockout of NLRP3 did not completely eliminate caspase-1 activation upon HCV infection. This result suggests other inflammasomes may be involved but have not yet been identified [22].

Other flaviviruses have also been shown to activate NlRP3, including Japanese Encephalitis Virus (JEV). Activation of NLRP3 by JEV occurred via potassium efflux and ROS production both in vitro and in vivo. This effect was dependent on viral replication, but the role played by the NLRP3 inflammasome in pathogenesis/viral clearance remains unclear [111]. A more recent study by Swaroop et al. revealed that knockdown of heat shock protein 60 resulted in decreased IL-1β production, which led to increased survival of JEV-infected mice [112], suggesting that the NLRP3 inflammasome may play a role in the pathogenesis of JEV. Interestingly, another recent study looked at the control of JEV in pigs, since pigs display less severe disease than other JEV hosts, and found no evidence of upregulation of NLRP3 inflammasome components or IL-1β. This may suggest the lack of NLRP3 activation serves as a protective effect in pigs and that overactivation of this inflammasome may contribute to the severe encephalitis documented in other hosts such as mice and humans [113]. We were unable to find any literature suggesting JEV activates any other inflammasomes.

One flavivirus that has been quite extensively studied in the context of NLRP3 activation is Dengue virus. This is of general interest because of the severe disease and immune dysfunction that is associated with secondary Dengue infection following a commonly mild primary infection. In a 2013 study, Wu et al. called attention to the fact that the exact trigger of caspase-1 and subsequent release of IL-1β during symptomatic Dengue infections was unknown despite occurring during human infection. The authors demonstrated that Dengue-infected inflammatory macrophages produced high levels of IL-18 and IL-1β, accompanied by pyroptosis, but this did not occur in resting macrophages. This suggests macrophages with inflammatory phenotypes, rather than resting macrophages, primarily contribute to the pathogenesis of Dengue infection [114]. Around the same time, it was shown that Dengue-induced NLRP3 inflammasome activation increased platelet-mediated endothelium permeability and this was followed by increased expression of IL-1β in vitro and in patient serum. The authors also showed that platelet shedding of IL-1β microparticles were correlated with increased vascular permeability [115]. Cheung et al. showed Dengue virus-2 infection of primary macrophages triggered activation of caspase-4 which subsequently induced activation of caspase-1 and secretion of IL-1β without the need for full inflammasome formation. Their data suggested that caspase-4 is upstream of caspase-1 and regulates inflammatory cytokine release in macrophages infected with Dengue-2 [116].

More recent research on Dengue-induced inflammasome activation has focused on determining what viral protein triggers NLRP3. Pan et al. showed that the dengue virus M protein could trigger the NLRP3 inflammasome along with secretion of IL-1β in mice which resulted in vascular leakage characteristic of Dengue Hemorrhagic Fever [19]. The M protein of Dengue was unable to induce vascular damage in NLRP3 knockout mice. The authors suggested that the Dengue M protein contributes to infection-associated vascular leakage [19]. Khan et al. determined that the E protein domain III (E III; immunodominant epitope) induced a pro-inflammatory signature that included secretion of IL-1β and TNF-α in THP-1 cells via the NF-κB pathway. The release of IL-1β was observed in conjunction with increased ROS production and K^+^ efflux. Then they showed the IL-1β production induced by E III was mediated by NLRP3 and caspase-1 and suggested this was responsible for Dengue-induced pathogenesis [117]. Further research by Lien et al. showed E III, which accumulates in plasma during viremia, induced hemorrhage and endothelial dysfunction in a manner dependent on the NLRP3 inflammasome in mice but was not seen when NLRP3 inhibitors were used during Dengue infection [118]. Injection of E III caused abnormalities in the endothelium. Injection of E III followed by injection of anti-Dengue NS1 antibodies caused more extensive vascular damage, liver dysfunction, and hemorrhage. When the same series of injections was given to NLRP3 knockout mice, disease was reduced compared to that of wild-type mice. The authors concluded that the NLRP3 inflammasome contributes to disease and may also be a therapeutic target for treating Dengue Hemorrhagic Fever [118].

A 2018 study by Fan et al. aimed to investigate the relationship between classical swine fever virus (CSFV) infection and inflammasome activation since many flaviviruses had already been shown to activate the NLRP3 inflammasome. The authors found that porcine peripheral blood mononuclear cells (PBMCs) infected with CSFV triggered the production of pro-IL-1β, its activation and maturation dependent on NLRP3 and caspase-1. They were also able to determine that knockdown of NLRP3 enhanced replication of CSFV, suggesting NLRP3 activation plays a role in the immune response to CSFV in swine monocytes [119].

#### 2.2.2. Pneumoviridae

Respiratory Syncytial Virus (RSV), a viral infection associated with particularly severe outcomes in young children, has been shown to activate the NLRP3 inflammasome, resulting in secretion of IL-1β [120,121]. This may indicate inflammasome activation contributes to the pathology associated with RSV infection. Notably absent is reported research on RSV induction of any other inflammasomes.

#### 2.2.3. Phenuiviridae

Another RNA virus that has been shown to fit the RNA-NLRP3 dichotomy is Rift Valley Fever Virus (RVFV). RVFV is an important pathogen as it can infect both livestock and humans, with livestock infections known to cause significant economic loses and lead to restrictions on trade, mostly in east and southern Africa [122]. There is a single paper [123] that documents activation of the NLRP3 inflammasome after infection with this virus. Research on this virus is restricted due to biosafety constraints requiring containment level (CL) 3 conditions. However, inflammasome activation by RVFV should be further studied to increase our understanding of this virus that can cause significant personal and economic losses for individuals living in underserved areas of the world.

#### 2.2.4. Filoviridae

Infection with Ebola causes substantial inflammation and immune dysregulation [124,125,126,127,128], so it seems likely that Ebola would trigger inflammasome activation. Using a CL2 virus-like particle system, Ebola was demonstrated to do just that, activating the NLRP3 inflammasome and inducing secretion of pyroptosis-associated cytokines in differentiated U-937 and THP-1 cells [129]. This study is the only report known to us that associates Ebola with inflammasome activation, and these results remain to be reproduced using a fully infectious virus system, which is understandably difficult due to both its classification as a Class A Select Agent and CL4 constraints [130].

#### 2.2.5. Coronaviridae

It has been documented that coronaviruses also activate their dogma-associated NLRP3 inflammasome. Several studies on SARS-CoV, prior to the emergence of SARS-CoV-2 and the onset of the COVID-19 (coronavirus disease 2019) pandemic, demonstrated that the viroporin 3a (ORF3a) [52,55], as well as the E protein, could trigger NLRP3 [131]. SARS-CoV ORF8b also triggers cellular stress pathways and activates the NLRP3 inflammasome [132]. Similarly, mouse hepatitis virus (MHV; another coronavirus) impairs the NLRP3 inflammasome, instead inducing inflammatory cell death mediated by caspase-8/RIPK3 [133].

#### 2.2.6. Rhabdoviridae

Lawrence et al. showed that rabies virus, a single-stranded, negative-sense RNA virus, upon infection of bone marrow-derived dendritic cells (BMDCs), resulted in production of pro-IL-1β which was cleaved into its mature form and released from cells using the NLRP3 inflammasome pathway. They also demonstrated that production of pro-IL-1β was dependent on viral replication and that IL-1 receptor-deficient mice were more seriously impacted by rabies infection, resulting in increased viral pathogenicity [134]. Kip et al. confirmed these results and suggested the inflammasome acts as an innate immune response to protect the host from the virus. Mice deficient in caspase-1 displayed increased disease severity when infected with an attenuated rabies strain (Evelyn-Rotnycki-Abelseth) [135], indicating the inflammasome is necessary for overcoming viral pathogenesis. Interestingly, mice deficient in IL-1β and IL-18 displayed the typical “mild symptoms” associated with the attenuated strain [135], suggesting these inflammatory cytokines do not contribute to the pathogenesis associated with rabies virus infection. By contrast, in 2019, it was reported that mice treated with a caspase-1 inhibitor displayed prolonged survival upon infection with rabies [18].

#### 2.2.7. Hantaviridae

The only known report of inflammasome activation by Hantaan virus, a negative-sense RNA virus in the *hantaviridae* family, found activation of the NLRP3 inflammasome along with secretion of IL-1β upon infection of THP-1 cells. The authors also linked ROS, but not ATP, release as a requirement for IL-1β production. They speculated that the NLRP3 inflammasome is responsible for increased levels of IL-1β in individuals suffering from hemorrhagic fever with renal syndrome induced by various hantaviruses [136].

#### 2.2.8. Paramyxoviridae

Several viruses from the *paramyxoviridae* family of negative-sense, single-stranded RNA viruses, are also known to activate and/or inhibit the NLRP3 inflammasome. Measles virus induces the NLRP3 inflammasome, resulting in secretion of IL-1β. By infecting cells with a mutant measles virus lacking the V protein, Komune et al. were able to show higher levels of IL-1β secretion compared to cells infected with wild-type virus. Using THP-1 cells stably expressing the V protein of measles, they were able to confirm this protein inhibits the secretion of IL-1β via inhibition of the NLRP3 inflammasome pathway [137].

We are aware of only a single paper that describes the interaction of Sendai virus (SeV) with the inflammasome pathway. Using THP-1 cells infected with a V gene knockout virus, Komatsu et al. showed that there was greater secretion of IL-1β than when those cells were infected with wild-type virus. NLRP3-deficient cells failed to generate such an increase in IL-1β when infected with the V gene knockout virus. They also found that the V protein inhibited formation of the inflammasome by blocking ASC oligomerization. Although the authors did not use fully infectious viruses, they were able to show that the V proteins of Nipah virus and parainfluenza virus type 2, other paramyxoviruses, inhibited NLRP3 complex formation and subsequent activation of IL-1β [138].

Wang et al. found that human macrophage-like THP-1 cells infected with Newcastle disease virus (NDV) induced IL-1β secretion via activation of the NLRP3 inflammasome using small RNA knockdown and NLRP3-deficient cells. Knockdown or chemical inhibition of inflammasome components showed increased cell survival upon NDV infection as well as increased replication [139]. Further investigation of NDV by Gao et al., from the perspective of understanding severe pathology in poultry infections, showed that IL-1β antibody treatment decreased body temperature and mortality following infection of chickens. They also showed that inhibition of NLRP3 or caspase-1 significantly reduced IL-1β expression and that NDV RNA alone was sufficient to induce IL-1β expression. These findings suggest that treatments targeting inflammasome pathway components may decrease disease associated with poultry infection [47].

## 3. Viruses That Do Not Fit the Dichotomy

### 3.1. DNA Viruses That Activate Traditional RNA Sensors

#### 3.1.1. Parvoviridae

Human bocavirus 1 (HBoV1) is a parvovirus that causes respiratory infections in young children. Unlike infections with other parvoviruses, HBoV1 has not been shown to activate apoptosis or necroptosis. However, when NLRP3 was inhibited, HBoV1-related cell death decreased. Therefore, NLRP3 seems to be the major receptor driving cell death (pyroptosis) during infection. Deng et al. also reported increased expression of IL-1α and IL-18 in infected cells. They showed that several viral proteins inhibit apoptosis and, in their absence, pyroptosis increased while viral replication was reduced [33]. Another 2017 paper showed that PBMCs taken from individuals with adult-onset Still’s disease (AOSD; an autoinflammatory condition), when compared to healthy controls, displayed elevated levels of NLRP3 mRNA and decreased expression of NLRP7, NLRP2, and NLRP12. Stimulation of PBMCs from AOSD patients with parvovirus B19 (which causes fifth disease, a mild rash that commonly affects children [140]) NS1 protein resulted in significant upregulation of NLRP3, caspase-1, and IL-1β transcript levels in comparison to PBMCs from healthy controls. This was accompanied by increases in supernatant levels of IL-1β and increased expression of NLRP3, IL-1β, and IL-18. These results suggest the B19 NS1 protein may induce inflammation associated with AOSD [65].

#### 3.1.2. Herpesviridae

Herpes Simplex Virus 1 (HSV-1) can cause oral or genital herpes and infects an estimated 3.7 billion people globally under the age of 50. There are currently no curative treatments or vaccines despite risk of more severe outcomes such as encephalitis, keratitis, or neonatal herpes, which can result in neurologic disability or infant death [141]. DNA viruses are inherently more complicated with regard to their innate immune response because of their large genomes and variety of accessory proteins (reviewed in [142,143,144]), and HSV-1 is no exception. In 2013, it was shown by Johnson et al. that HSV-1 induced activation of both the IFI16 and NLRP3 inflammasomes, accompanied by mature IL-1β secretion in vitro [34]. Interestingly, IFI16 recognized the genome of HSV-1 in infected cells prior to viral gene expression, but later in infection, HSV-1 targeted IFI16 for degradation, thereby inhibiting maturation of IL-1β. In contrast, there was no obvious targeting or decrease in NLRP3 or ASC [34]. Following up on IFI16 degradation, the same group found that IFI16 plays an important role in regulating HSV-1 replication. When IFI16 was knocked down, HSV-1 production increased six-fold, and when IFI16 was overexpressed, HSV-1 production decreased five-fold [61]. They further showed that IFI16 can restrict HSV-1 replication in several ways, including by IFI16 molecule accumulation on the genome itself, repressing expression of viral genes, and by directly or indirectly modulating histone modifications [61].

Maruzuru et al. called attention to the fact that the role of inflammasomes during HSV-1 infection in vivo is less well understood than in vitro. They showed that HSV-1 VP22 interacts with AIM2, preventing its oligomerization. An HSV-1 mutant lacking VP22 (HSV-1 ΔVP22) was shown to activate AIM2, which subsequently led to secretion of IL-1β and IL-18. Secretion of these cytokines did not happen in absence of AIM2. Additionally, the ΔVP22 mutant showed diminished viral replication but was restored in AIM2-deficient mice [62]. The mechanism of inflammasome activation by HSV-1 in human macrophages is unknown. Using THP-1 cells with various components of the inflammasome pathway knocked out, Karaba et al. showed that HSV-1 inflammasome activation in macrophages is dependent on NLRP3, ASC, and caspase-1, but not AIM2 or IFI16. NLRP3 inflammasome activation in macrophages resulted in secretion of IL-1β but not IL-18. Interestingly, inactivated HSV-1 enhanced inflammasome activation, suggesting that viral replication of HSV-1 suppresses inflammasome activation under normal conditions [63]. The activation of various inflammasome sensors by herpesviruses is comprehensively reviewed in a recent publication by Kumar et al. [64].

#### 3.1.3. Hepadnaviridae

The pathophysiology of hepatitis B virus (HBV) remains unclear but it has been suggested that liver disease, in various contexts, is mediated by the inflammasome (reviewed in [145]). Further HBV investigation is important considering that, despite availability of an effective vaccine, more than 820,000 people die every year due to complications from chronic infection [146]. In 2015, Han et al. showed that the expression of AIM2 in individuals with chronic HBV was significantly higher than in individuals with chronic HCV, and the expression of AIM2 was even higher in individuals with high amounts of HBV replication. Expression of AIM2 was also correlated with inflammatory activity, levels directly correlated with expression of caspase-1, IL-1β, and IL-18 in HBV-infected livers [68]. To further the understanding of the role of inflammasomes in HBV infection, Askari et al. considered expression of various inflammasome markers in monocytes from individuals infected with HBV. They showed expression levels of NLRC4, NLRP3, and NLRP1 in circulating monocytes were not significantly different between HBV-infected individuals and healthy controls. mRNA levels of these markers were also not significantly different, regardless of HBV DNA copy number [67].

Since the role of the inflammasome during infection with HBV was still unclear, Chen et al. investigated mRNA expression levels of AIM2, IFI16, and caspase-1 in PBMCs from study subjects with acute or chronic HBV infections. In both groups, inflammasome-associated genes were upregulated. Chronically infected individuals displayed mRNA levels of AIM2 and IFI16 that were significantly positively correlated with serum HBV load. Only individuals with acute HBV had increased serum levels of IL-1β and IL-18. The authors found no evidence of NLRP3, AIM2, or IFI16 inflammasome activation in chronically infected individuals. In vitro stimulation of PBMCs from chronically infected individuals showed activation of AIM2 and IFI16 but this could be suppressed by HBeAg. Taken together, the results suggest activation of the AIM2 and IFI16 inflammasomes (but not NLRP3) during acute HBV infection. However, it appears that the AIM2 and IFI16 inflammasomes can be inhibited by HBeAg during chronic infection and may contribute to HBV-induced immunotolerance [66]. To follow up on this research [66], Ding et al. were interested in the role of the HBcAg during inflammasome activation in the context of HBV infection. They showed that, when HepG2 cells were stimulated with LPS and transfected with HBcAg, NLRP3 activation and IL-1β production was promoted. However, this was not seen when HBcAg and HBeAg were transfected together. The authors concluded that HBV core antigen may contribute to liver inflammation associated with chronic HBV infection [53].

More recent studies have moved towards an even greater understanding of the role of inflammasomes during HBV infection. A study by Xie et al. aimed to investigate the role of the HBV X protein under conditions of oxidative stress. To do this, they stimulated HL7702 liver-derived cells with hydrogen peroxide and transfected them with the HBV X protein. They showed the X protein triggered the release of ASC, IL-1β, and IL-18. The authors were able to correlate levels of NLRP3, ASC, and IL-1β with HBV DNA concentration in tissues collected from HBV-infected individuals [35]. Since HBV has been shown to change the phenotype of macrophages to induce either M1-like pro-inflammatory or M2-like anti-inflammatory polarization, Li et al. showed M1-like macrophages exhibited a strong HBV-suppressive response that was not seen in M2-like macrophages. M1-like macrophages, stimulated by HBV, triggered expression of IL-1β. Multiple HBV proteins were able to induce IL-1β expression in macrophages. Macrophages responded to HBV infection by expressing IL-1β which may aid in suppression of HBV replication [69]. Despite extensive research on HBV inflammasome activation, further research is necessary in order to fully understand the pathology associated with chronic HBV infection.

#### 3.1.4. Adenoviridae

Adenoviruses are one of the causes of the common cold [147] but also have great therapeutic potential for both their use as vector vaccine backbones as well as a delivery system for gene therapy [148,149,150]. For both reasons, understanding innate immune responses, including that of inflammasomes, against adenoviruses is essential. Barlan et al. showed that secretion of IL-1β by adenovirus type 5 (Ad5) infection of macrophages is dependent on NLRP3, ASC, and caspase-1. They showed that induction of IL-1β is dependent on toll-like receptor 9 (TLR9) which senses double-stranded DNA (like that of adenoviruses). A temperature-sensitive mutant of Ad5 (that cannot penetrate endosomal membranes) was unable to induce IL-1β secretion, suggesting endosomal membrane penetration is required for NLRP3 inflammasome activation by Ad5. They also showed that Ad5 entry induces ROS and that ROS inhibitors prevent IL-1β secretion [36]. Schulte et al., being particularly interested in the immune responses to adenoviruses, showed cytokine induction occurred after internalization of viral DNA into the cytoplasm. When they inhibited AIM2 or NLRP3, cytokine response was decreased [72]. Continuing this research, Eichholz et al. showed that there is direct engagement of the adenoviral genome with AIM2 which subsequently engages ASC. This leads to caspase-1 activation, accompanied by IL-1β and GSDM-D cleavage in dendritic cells [70]. It was shown that Ad5 infection of THP-1 macrophages resulted in increased secretion of cleaved caspase-1 and IL-1β. Using an Ad5 mutant virus that was defective in the expression of virus-associated (VA) RNAi (small non-coding RNAs), Darweesh et al. showed NLRP3 activation which led to ASC speck formation and increased cell lysis. In vitro synthesized VA RNAI was able to inhibit NLRP3 activation in THP-1 cells, suggesting that viral VA RNAI is able to inhibit NLRP3 [71].

### 3.2. RNA Viruses That Activate Traditional DNA Sensors

#### 3.2.1. Flaviviridae

We have already discussed several flaviviruses that fit the RNA-NLRP3 dichotomy. However, there are also several that do not fit the dichotomy. In 2018, it was reported that substantial inflammasome activation was present in the central nervous system of fatal Zika virus microcephaly cases. In tissue samples from fatal neonatal cases positive for Zika virus, increased expression of NLRP1, NLRP3, and AIM2 was reported when compared to flavivirus-negative controls [37]. It is unknown at this time whether inflammasome activation is involved in Guillain-Barré syndrome, another known complication of Zika infection.

Since the first detection of West Nile Virus (WNV) in North America in 1999, The United States’ Centers for Disease Control and Prevention has reported 52,532 human cases with 25,849 documented to have neuroinvasive disease [151]. Interestingly, before WNV was shown to activate the traditional NLRP3-RNA virus sensor, it was shown to activate the RIG-I inflammasome, which had antiviral effects in conjunction with caspase-12. Using caspase-12 knockout mice, these authors showed that, upon infection with WNV, there was significantly more mortality, higher viral load, and a less effective type I IFN response compared to that of wild-type mice. Primary neurons and embryonic fibroblasts from mice were infected with WNV and showed that caspase-12 positively mediated the type I IFN response through its regulation of TRIM25 (E3 ubiquitin ligase) which subsequently mediates the ubiquitination of RIG-I [73].

To further understand the role of the inflammasome in the central nervous system (CNS) during WNV infection, Ramos et al. used a mouse model to help elucidate the role of IL-1β since it had been shown to be elevated in plasma of infected individuals. The authors showed that host IL-1β increases in infected animals with both IL-1β and NLRP3 acting as restriction factors to control viral infection in the CNS. Mouse models that were lacking the IL-1 receptor or components of the inflammasome pathway were more susceptible to WNV-induced pathogenic effects, including accumulation of virus in the CNS but not in peripheral tissues. The authors also noted signs of decreased quality of CD8^+^ T cell responses and reduced overall antiviral activity in the CNS. They did show that cortical neurons secrete IL-1β in response to WNV and that IL-1β “synergizes” with type I IFN to suppress replication of WNV in neurons. The authors concluded that the NLRP3 inflammasome pathway is critical in the control of WNV infection in the CNS and that IL-1β can aid in restriction of viral replication of WNV in neurons [74].

Due to the inflammatory nature of WNV and its association with encephalitis, Kumar et al. wanted to further investigate the role of the inflammasome in the pathogenesis of WNV infection. They showed that ASC was necessary for IL-1β production, caspase-1 activation, and for an effective host response to generate immunity to WNV. ASC-deficient mice showed increased susceptibility to WNV, accompanied by decreased survival which was associated with increased viral replication in peripheral and CNS tissues [75]. The authors report that ASC knockout mice displayed decreased levels of inflammatory cytokines in serum accompanied by an interesting finding of reduced IFN-α and IgM in serum. However, brains of ASC knockout mice showed “unrestrained inflammation,” including elevated levels of IFN-γ, CCL2, and CCL5. This increase in chemokine levels was also associated with activation of astrocytes and increased infiltration of peripheral immune cells into the CNS along with increased death of neurons. The authors conclude that ASC is necessary for modulating inflammasome-dependent immune responses and that its activation is necessary for clearance of WNV [75].

Ekchariyawat et al. were interested in the similarity of innate immune responses to arboviruses in general so they examined the effect of both Chikungunya (CHIKV) and WNV on the inflammasome pathway. They found that both viruses induced secretion of IL-1β and maturation of caspase-1 in resident skin cells. They also showed activation of AIM2 by both viruses in dermal fibroblasts, the first report of WNV activating a traditionally associated DNA sensor. siRNA knockdown of AIM2 and caspase-1 led to decreased secretion of IL-1β from infected cells. In contrast to their findings regarding CHIKV, which will be discussed later, caspase-1 inhibition did not affect replication of WNV. This suggests the inflammasome is not directly involved in the pathogenesis or immunological clearance of WNV [76].

#### 3.2.2. Coronaviridae

SARS-CoV-2, the causative agent of COVID-19, has been associated with substantial inflammation and over-activation of the immune system in severe cases [152,153,154,155]. In contrast to SARS-CoV, SARS-CoV-2 NS proteins 1 and 13 were found to suppress activation of the NLRP3 inflammasome [156]. However, the SARS-CoV-2 N protein induced hyperinflammation via the NLRP3 inflammasome, with the implication that overactivation may contribute to the pathology associated with severe COVID-19 [157]. This finding was confirmed by the inhibition of NLRP3 by MCC950, which resulted in lower levels of inflammatory cytokine release, and the use of NLRP3 knockout mice infected with SARS-CoV-2, which displayed less severe disease [155]. Besides viral proteins, single-stranded RNA derived from SARS-CoV and SARS-CoV-2 was reported to trigger TLR-8, leading to secretion of IL-1β in absence of pyroptosis. Campbell et al. showed that activation of IL-1β was dependent on caspase-1, caspase-8, NLRP3, and K^+^ efflux, describing this as a non-classical inflammasome pathway [158]. Even more recently, it was demonstrated that the inflammasome pathway in monocytes is activated upon abortive infection with SARS-CoV-2 which was suggested to drive the severe inflammatory pathology associated with severe cases of COVID-19 [10].

#### 3.2.3. Picornaviridae

Similarly, Enterovirus A71 (EV-A71), the causative agent of hand-foot-and-mouth disease, has been shown to activate both RNA- and DNA-associated inflammasomes. A 2017 paper reported that AIM2 was activated in both cell-culture infected cells and in the CNS of fatal cases of EV-A71 encephalomyelitis [77]. Unsurprisingly, considering the present discussion, a previous report had already indicated activation of the NLRP3 inflammasome upon infection with EV71 [23]. Although at odds with reported research on Zika, both EV71 findings were associated with decreased viral replication, indicating inflammasome activation plays a role in protection against severe EV71 infection.

#### 3.2.4. Togaviridae

Mayaro virus (MAYV) is a positive-sense, single-stranded RNA virus that circulates in South America and is transmitted to humans via mosquitos [159]. Like many other emerging viral pathogens, many aspects of MAYV remain understudied, including the human immune response. A study by de Castro-Jorge et al. established a model of MAYV infection in macrophages and mice. Using these models, they showed that inflammasome pathway components (NLRP3, ASC, AIM2, and caspase-1) are induced upon infection of bone marrow-derived macrophages. Using NLRP3 knockout mice, they showed that NLRP3, but not AIM2, was required for secretion of IL-1β. Confirming the role of inflammasome activation, the authors reported increased levels of caspase-1-p20, IL-1β, and IL-18 in serum of individuals infected with MAYV in comparison to that of healthy controls [20].

CHIKV is spread by mosquitos and can cause severe muscle aches, joint pain, headache, fever, and rash [160]. As mentioned above, Ekchariyawat et al. showed that both CHIKV and WNV activate inflammasomes, accompanied by maturation of both caspase-1 and IL-1β, and that AIM2 was involved in this process. To analyze the effect of inflammasome activation on viral replication, they used silencing to inhibit caspase-1, which had no effect on WNV, but did show improved replication of CHIKV in dermal fibroblasts. This suggests the pro-inflammatory microenvironment of the skin is helpful in mitigating disease and important for virus control, at least in the context of CHIKV infection [76]. In another study by Chen et al., it was shown that CHIKV infection activated the NLRP3 inflammasome in both humans and mice. PBMCs from infected individuals displayed elevated NLRP3, caspase-1, and IL-18 mRNA expression. Using a mouse model, they showed that NLRP3 infection was associated with high levels of inflammatory symptoms. Using MCC950 (NLRP3 inhibitor), they observed decreased inflammation, abrogated osteoclastogenic bone loss, and myositis associated with infection. However, there was no effect on viral replication. Mice treated with MCC950 also showed decreased levels of IL-6, chemokine ligand 2, and TNF in joint tissue [78]. Together, these findings suggest inflammasome activation by CHIKV is involved in the inflammatory disease state associated with infection.

#### 3.2.5. Orthomyxoviridae

Influenza-induced pyroptosis may be the most well studied of all forms of virus-induced inflammasome activation and pyroptosis. Although outside the scope of this review, it is important to consider that the role of NLRP3 inflammasome activation by influenza is highly debated within the field. Some groups report it as a mechanism of pathogenesis [16,17,81,82] while others report it as necessary for an effective adaptive immune response to clear influenza infection [5,25,26,83]. Besides NLRP3, influenza has also been shown to activate RIG-I (which promotes replication) [5] and AIM2. Activation of these inflammasomes is associated with worse prognosis in infected mice without influencing viral load [38]. Recently, it was shown that IFI16 is activated directly by influenza RNA, causing an enhancement of RIG-I transcription and activation, potentially leading to restriction of influenza infection [85]. It has been proposed that influenza proteins M2 [15] and PB1-F2 [25] can activate NLRP3, alter ion balance, and extensively affect cell homeostasis. These changes can result in release of oxidized DNA which, in turn, can activate AIM2, demonstrating influenza indirectly activates this DNA-associated sensor [86]. Another group showed that DNA in the microenvironment of the lung during influenza infection can trigger AIM2, adding to the research suggesting pyroptosis and inflammasome activation is a circular process [79]. Recently, influenza has been shown to activate NLRC4 which regulates influenza-specific T cells [80]. Influenza viruses H5N1 and H3N2 with accessory protein PB1-F2 have also been shown to limit NLRP3-NEK7 complex formation and subsequent pyroptosis. Mutant viruses lacking PB1-F2 induced higher levels of cleaved-caspase-1, cleavage of GSDM-D, and release of lactate dehydrogenase (LDH) and IL-1β. PB1-F2 was shown to limit transition of NLRP3 from its closed confirmation into its active state [87]. On the opposite side is the research that has shown that the NS1 protein of influenza can inhibit the NLRP3 inflammasome [83]. The extensive body of literature that focuses on inflammasome activation by influenza viruses is invaluable and will certainly guide future work on inflammasome activation by other viruses.

#### 3.2.6. Retroviridae

HIV causes disease by depleting CD4^+^ T cells, rendering the host incapable of fighting off other opportunistic infections. Early studies suggested CD4^+^ T cells died by apoptosis [161] (reviewed in [162]). However, in 2014 it was shown that the uninfected T cells that die are actually abortively infected with HIV and die via pyroptosis in a caspase-1-dependent manner, accompanied by IL-1β secretion [89]. Around the same time, the same research group published their findings that IFI16, another traditionally DNA-associated sensor, was required for the pyroptotic death of CD4^+^ T cells. They suggested that the IFI16 inflammasome is likely a driver in the development of AIDS [90]. Further research confirmed these findings, showing that individuals with HIV had more CD4^+^ T cells that were ASC^+^ than healthy controls [91]. Since then, HIV has also been shown to activate NLRP3 in the context of CD4^+^ T cell loss [92] as well as HIV-induced neuropathy [93]. Given the replication cycle of this virus, including its use of DNA intermediates, it is not surprising that HIV activates sensors typically associated with both RNA and DNA viruses. At the present time, we are unaware of any publications that show activation of the AIM2 inflammasome during HIV infection. 

## 4. Other Inflammasomes

This review illustrates that two main inflammasome sensors have been well studied in the context of viral infection: NLRP3 and AIM2 (summarized in Table 1). Other inflammasomes that have been identified as being activated by various viruses include, but are not limited to, IFI16, NLRC4, NLRP1, NLRP9b, and NLRP12. Research aimed at understanding the activation and/or inhibition of these other inflammasomes is generally limited to only a couple of studies for each sensor, meaning the extent to which these inflammasomes are activated by infection with various viruses remains unclear. Below are a few examples of viral activation of other inflammasomes. This is by no means an exhaustive list but does highlight the need for more research into viral induction of inflammasome sensors other than NLRP3 and AIM2.

The 3C protease of enteroviruses (such as rhinoviruses that cause the common cold) has been shown to activate NLRP1 in human airway epithelial cells [163]. Robinson et al. were able to show that the 3C protease directly cleaves NLRP1 which leads to inflammasome formation and IL-18 secretion, suggesting NLRP1 may play a role in the pathogenesis of viral infections of the respiratory tract [163].

The NLRC4 inflammasome has been shown to play diverse roles during both rotavirus and influenza virus infections. NLRC4 activation during rotavirus infection leads to the release of IL-18 and IL-22. IL-22 release induced a state of gene expression in intestinal epithelial cells that was protective against rotavirus infection, while IL-18 aided in the elimination of rotavirus-infected cells [164]. A study of immune responses to influenza virus showed that NLRC4 activation in dendritic cells was necessary for virus-specific CD4^+^ T cell responses. Using a mouse model, the authors showed that NLRC4 knockout mice had decreased survival rates and increased viral titers accompanied by normal CD8^+^ T cell responses and severely impaired CD4^+^ T cell responses [80].

A paper from late 2021 identified activation of the NLRP6 inflammasome during various viral infections, including SARS-CoV-2, rotavirus, and MHV. The authors report that NLRP6 is activated in the lungs of pneumonia patients with severe COVID-19 as well as in hepatocytes of mice in response to MHV infection. Interestingly, they determined the activation of NLRP6 is dependent on dsRNA, suggesting coronavirus replication intermediates may trigger this inflammasome [88].

In a 2018 study by Zhu et al., the authors showed that NLRP9b restricts rotavirus replication in intestinal epithelial cells which subsequently leads to the maturation of IL-18 and GSDM-D-mediated pyroptosis of infected cells. Depletion of NLRP9b or other downstream components of the inflammasome pathways in mice caused more severe disease and enhanced susceptibility to rotavirus infection. The authors also determined that NLRP9b recognizes dsRNA with the help of RNA helicase Dhx9 [96].

NLRP12, conversely to NLRC4, has been shown to play a role in detrimental immune cell recruitment during IAV infection. Using NLRP12 knockout mice infected with IAV, Hornick et al. showed that there was reduced vascular permeability and fewer pulmonary neutrophils compared to wild type mice. These findings suggest NLRP12 plays a role in the pathogenesis of IAV infection, possibly by mediating adverse neutrophil recruitment to the respiratory tract [165].

Many other inflammasome sensors have been described but the role, if any, they play during viral infection remains to be determined.

## 5. Addressing the Dichotomy: What Perpetuates This Dichotomy?

By reviewing current research, it has become clear that many viruses activate multiple inflammasomes. Of particular interest is the fact that, in every case that a virus is reported to have activated an inflammasome that does not align with the generally accepted dogma, the particular virus has also been shown to activate the sensor that does correspond with the associated dogma. What can we conclude? Here we speculate that it is likely that many, if not all, viruses activate multiple inflammasomes, but there is little conclusive evidence because the activation of multiple inflammasomes by the same virus has rarely been addressed in the existing literature. As scientists, we tend to begin experiments with a hypothesis that is usually influenced by previous experience, literature, dogma, etc. We propose here that this implicit bias may explain why few traditionally RNA-associated sensors have been reported as being activated by DNA viruses, and vice versa, few papers in the literature report that RNA viruses can activate traditionally DNA-associated sensors.

Biosafety constraints are likely one major barrier to studying inflammasome activation by some viruses. Rightly so, most research performed under CL3 or CL4 conditions focuses on vaccines and/or antivirals, leaving little time for classic basic science research driven by curiosity. It seems likely that many CL3 and CL4 viruses would induce multiple inflammasomes as most of these viruses are associated with substantial inflammation. Simple searches for inflammasome-related terms and viruses such as Crimean-Congo hemorrhagic fever, Nipah, Marburg, Lassa fever virus, monkeypox, or various other hemorrhagic arenaviruses reveal no scientific publications. There is an obvious gap in our understanding of CL4 agents and their abilities to inhibit or activate inflammasomes despite the potential of such research to aid in treatment development and/or clinical management of these viral infections.

There seems to exist a general bias in the scientific literature that RNA viruses activate NLRP3 while DNA viruses activate AIM2 or IFI16. Here, we highlight existing evidence that many viruses do not fit this dogma and encourage the scientific community to follow up on and expand our knowledge regarding the activation of multiple inflammasome sensors by a single virus. We propose that more time and effort be invested in furthering our understanding of virus-induced inflammasome activation, breaking down the existing false dichotomy in the process. Another consideration that requires the attention of inflammasome researchers is the expanded publication of negative results as these data could aid significantly in our understanding of whether the promiscuity of inflammasome activation is indeed widespread or more constrained to specific conditions.

## 6. Conclusions

It is clear that activation of the NLRP3 inflammasome by RNA viruses and activation of the AIM2 inflammasome by DNA viruses has been fairly well-studied. However, research on activation of inflammasomes by their oppositely-associated viruses is lacking. Virus-induced pyroptosis literature continues to grow, but significant gaps remain apparent, including the exact trigger of NLRP3. Additionally, many inflammasomes have been identified, but the role they play during viral infection requires further study. We, as scientists, should take a broader view when looking for “classic” RNA- or DNA-associated sensors within a respective system which should include the practice of publishing any relevant negative results. With some extra time and effort, we should have a well-rounded picture of the promiscuity of inflammasome activation, including the role of inflammasomes in the pathogenesis of viral infections.

## Figures and Tables

**Table 1:** Viruses, separated by family, with inflammasome sensors activated and references associated with each one.

Family	Virus	Inflammasome Sensor(s) Activated	Against Dichotomy?	Reference(s)
*Poxviridae*	Vaccinia	AIM2	no	[39]
*Papillomaviridae*	HPV	AIM2, IFI16	no	[101,102]
*Herpesviridae*	CMV	AIM2	no	[32]
	HSV-1	NLRP3, AIM2, IFI16	yes	[34,61,62,63,64]
	KSHV	IFI16	no	[104]
*Parvoviridae*	HBoV1	NLRP3	yes	[33,65]
*Hepadnaviridae*	HBV	AIM2, NLRP3, NLRP1, NLRC4, IFI16	yes	[35,53,66,67,68,69]
*Adenoviridae*	Ad5	NLRP3, AIM2	yes	[36,70,71,72]
*Flaviviridae*	HCV	NLRP3	no	[22,94,105,106]
	JEV	NLRP3	no	[111,112,113]
	Dengue	NLRP3	no	[114,115,116,117,118]
	CSFV	NLRP3	no	[119]
	Zika	NLRP1, NLRP3, AIM2	yes	[37]
	WNV	RIG-I, NLRP1, NLRP3, AIM2	yes	[73,74,75,76]
*Pneumoviridae*	RSV	NLRP3	no	[120,121]
*Phenuiviridae*	RVFV	NLRP3	no	[123]
*Filoviridae*	Ebola	NLRP3	no	[129]
*Coronaviridae*	SARS-CoV	NLRP3	no	[52,55,131,132,158]
	SARS-CoV-2	NLRP3, AIM2, NLRP6	yes	[10,88,155,156,157,158]
	MHV	NLRP3	no	[133]
*Rhabdoviridae*	Rabies	NLRP3	no	[18,134,135]
*Hantaviridae*	Hantaan	NLRP3	no	[136]
*Paramyxoviridae*	Measles	NLRP3	no	[137]
	SeV	NLRP3	no	[138]
	NDV	NLRP3	no	[47,139]
*Picornaviridae*	EV71	NLRP3, AIM2	yes	[23,77]
*Togaviridae*	Mayaro	NLRP3, AIM2	yes	[20]
	CHIKV	AIM2, NLRP3	yes	[76,78]
*Orthomyxoviridae*	IAV	NLRP3, RIG-I, IFI16, AIM2	yes	[5,15,16,17,25,26,38,79,80,81,82,83,84,85,86,87]
*Retroviridae*	HIV	NLRP3, IFI16	yes	[89,90,91,92,93]

## Data Availability

Not applicable.
